# Mechanical Abnormalities of the Airway Wall in Adult Mice After Intrauterine Growth Restriction

**DOI:** 10.3389/fphys.2019.01073

**Published:** 2019-08-23

**Authors:** Peter B. Noble, Darshinee Kowlessur, Alexander N. Larcombe, Graham M. Donovan, Kimberley C. W. Wang

**Affiliations:** ^1^School of Human Sciences, University of Western Australia, Perth, WA, Australia; ^2^Telethon Kids Institute, University of Western Australia, Perth, WA, Australia; ^3^School of Public Health, Curtin University, Perth, WA, Australia; ^4^Department of Mathematics, University of Auckland, Auckland, New Zealand

**Keywords:** intrauterine growth restriction, low birth weight, animal models, asthma, respiratory structure and function

## Abstract

Developmental abnormalities of airways may impact susceptibility to asthma in later life. We used a maternal hypoxia-induced mouse model of intrauterine growth restriction (IUGR) to examine changes in mechanical properties of the airway wall. Pregnant BALB/c mice were housed under hypoxic conditions (10.5% O_2_) from gestational day (GD) 11 to GD 17.5 (IUGR; term, GD 21). Following hypoxic exposure, mice were returned to a normoxic environment (21% O_2_). A control group of pregnant mice were housed under normoxic conditions throughout pregnancy. At 8 weeks postnatal age, offspring were euthanized and a tracheasectomy performed. Tracheal segments were studied in organ baths to measure active airway smooth muscle (ASM) stress to carbachol and assess passive mechanical properties (stiffness) from stress-strain curves. In a separate group of anesthetized offspring, the forced oscillation technique was used to examine airway mechanics from relative changes in airway conductance during slow inflation and deflation between 0 and 20 cmH_2_O transrespiratory pressure. From predicted radius-pressure loops, storage and loss moduli and hysteresivity were calculated. IUGR offspring were lighter at birth (*p* < 0.05) and remained lighter at 8 weeks of age (*p* < 0.05) compared with Controls. Maximal stress was reduced in male IUGR offspring compared with Controls (*p* < 0.05), but not in females. Sensitivity to contractile agonist was not affected by IUGR or sex. Compared with the Control group, airways from IUGR animals were stiffer *in vitro* (*p* < 0.05). *In vivo*, airway hysteresivity (*p* < 0.05) was increased in the IUGR group, but there was no difference in storage or loss moduli between groups. In summary, the effects of IUGR persist to the mature airway wall, where there are clear abnormalities to ASM contractile properties and passive wall mechanics. We propose that mechanical abnormalities of the airway wall acquired through disrupted fetal growth impact susceptibility to disease.

## Introduction

The early life presentation of lung function impairment in asthma implicates a developmental disorder as the driver for disease. Reduced airway function (Turner et al., 2004) and exaggerated airway narrowing to bronchial challenge (Palmer et al., 2001) is reported in infants who go on to develop asthma in childhood, a relationship which has now been shown to extend to adulthood (Owens et al., 2017). These findings are also consistent with early deficits in FEV_1_ in children with asthma (Bisgaard et al., 2012) with any decline in lung function in later life differing only marginally from the healthy lung (Phelan et al., 2002; Sears et al., 2003; James et al., 2005). Impaired respiratory function may therefore be a risk factor for asthma rather than a consequence of ongoing pathological processes.

At a population level, asthma development is linked with a number of prenatal events that include intrauterine growth restriction (IUGR) (Källén et al., 2013) and low birth weight (Barker et al., 1991). Working on the premise that such associations were mediated by persisting functional changes to the airway, we established a mouse model of maternal hypoxia-induced IUGR to show that IUGR female offspring were hyperresponsive to methacholine and male IUGR offspring hyporesponsive at 8 weeks of age (Wang et al., 2018). Functional abnormalities of the adult airway were therefore the result of an *in utero* insult, specifically corresponding to the pseudoglandular-canalicular period when the airway develops. Further, sex-dependent effects of IUGR aligned well with differences in the prevalence of asthma between males and females; hyperresponsiveness in adult females and hyporesponsiveness in adult males are broadly consistent with greater prevalence in adult females (Schatz and Camargo, 2003).

Determining the mechanism through which an *in utero* insult can affect function of the adult airway is important as this represents a physiological determinant of future disease. However, the cause of above changes in airway function after IUGR and low birth weight (Wang et al., 2018) could not be established. Given that airway responsiveness is strongly impacted by airway wall structure (Noble et al., 2013) and lung volume (Ding et al., 1987), these properties were initially assessed; no differences were found in either wall thickness [including airway smooth muscle (ASM)] or plethysmographically determined lung volume (Wang et al., 2018). Other than wall thickness, changes in “contractility” of the ASM (force for a given cross section area) theoretically impacts airway narrowing capacity, as has been documented in subjects with fixed airflow obstruction (Opazo Saez et al., 2000). Airway stiffness may also affect airway responsiveness by blunting protective bronchodilatory effects of breathing stresses (Noble et al., 2007).

This study examined previously unassessed mechanical properties of the airway wall from IUGR-affected adult mice including ASM contractility and airway stiffness. Male and female offspring were studied in order to determine whether sex differences in airway responsiveness *in vivo* are explained by intrinsic changes to the airway wall. Findings suggest that changes in ASM contractility and passive mechanical properties contribute to functional abnormalities observed in adulthood after IUGR.

## Materials and Methods

### Ethical Approval

Our experimental approach was to subject pregnant mice to a hypoxic or normoxic environment and, in both male and female offspring, to measure mechanical properties of the ASM and airway wall. This study was carried out in strict accordance with the recommendations of the Australian Code of Practice for the Care and Use of Animals for Scientific Purposes (7th Edition). The protocol was approved by the Telethon Kids Institute Animal Ethics Committee (Project Number 264).

### Maternal Hypoxia-Induced Intrauterine Growth Restriction Mouse Model

Pregnant female BALB/c mice at gestational day (GD) 7 were obtained from Animal Resources Centre (Murdoch, WA, Australia) and housed at the Telethon Kids Institute in specific pathogen-free environments. Mice were maintained on a 15:9-h light:dark cycle and supplied with an allergen-free diet (Specialty Feeds, Glen Forrest, WA, Australia) and water *ad libitum*. One group of pregnant mice were housed under hypoxic conditions (10.5% O_2_) from GD 11 to GD 17.5 (pseudoglandular-canalicular stage of mouse lung development) and then returned to a normoxic environment (21% O_2_) until birth (GD 21) (Wang et al., 2018). A control group of pregnant mice was housed under normoxic conditions throughout pregnancy (Wang et al., 2018). Weights of offspring were recorded at birth and 8 weeks of age, prior to *in vitro* organ bath experimentation. Mice were euthanized by overdose i.p. ketamine and xylazine. Tracheasectomy was performed and tracheal segments placed immediately into chilled Krebs solution (Ansell et al., 2014).

### Organ Bath Studies

A DMT myography system (620 M) was used for the study of tracheal segments. Organ bath chambers contained heated Krebs solution gassed with carbogen (95% O_2_:5% CO_2_) (Ansell et al., 2014; Cairncross et al., 2018). The dissected tracheal segment (~2.5 mm in length) was slid onto two stainless steel prongs, one connected to a transducer and the other to a micrometer (Cairncross et al., 2018). Posterior ASM was aligned in series with the transducer. The micrometer was adjusted to distend the tracheal lumen diameter (and therefore ASM). Lumen “diameter” was approximated by the external distance between the prongs, determined under a dissecting microscope and calibrated to a 1-mm graticule. An initial force of 0.2 g was maintained over a 1-min period which was sufficient to ensure that the tissue was held fixed in position. Initial lumen diameter was denoted *D*_i_ and was not different between groups (data not shown).

**Figure 1:**
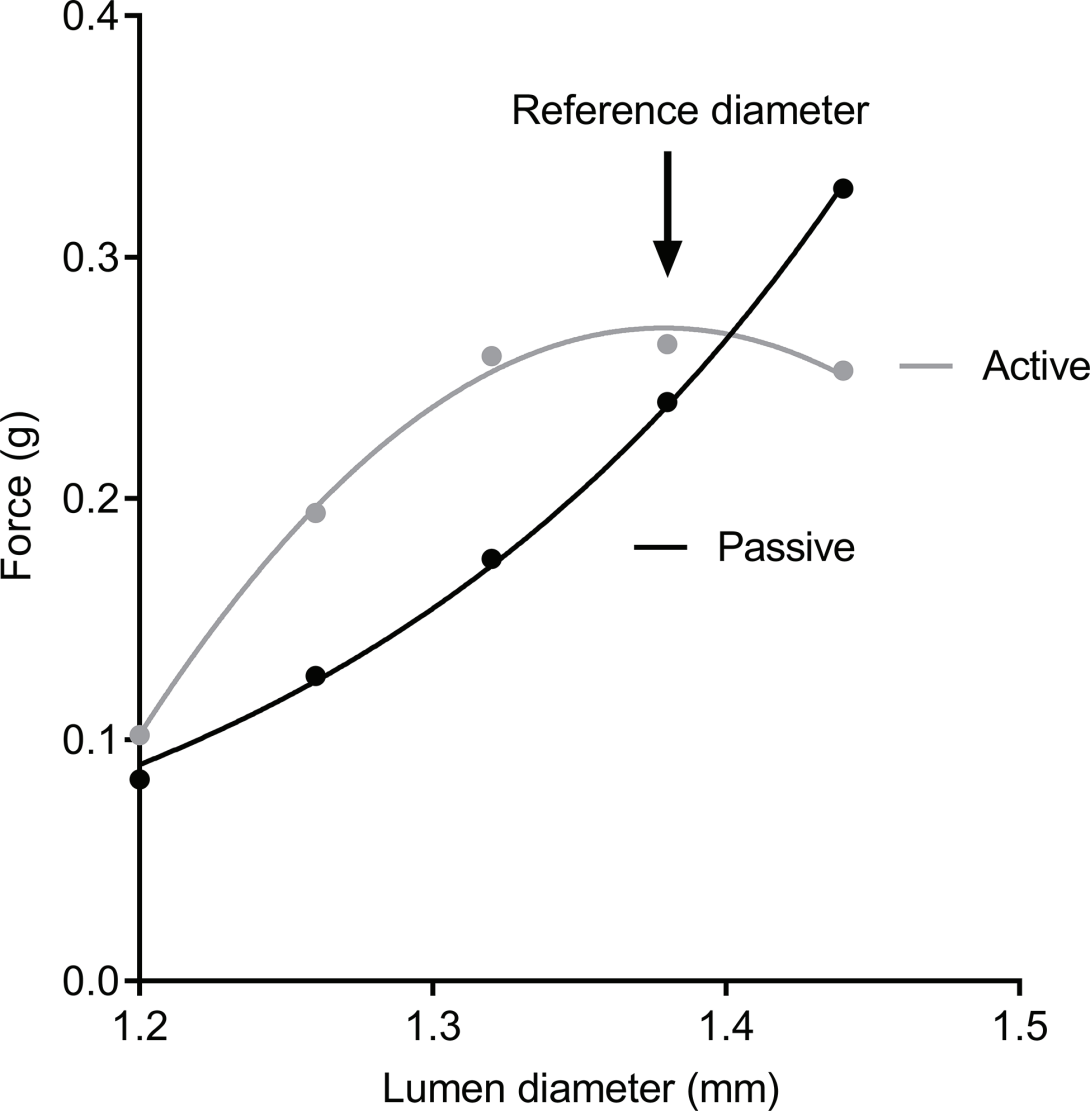
Diameter-force curves. Passive force (black circles) and active force (gray circles) after stimulation to KCl solution. Peak contraction was used to establish a reference diameter for dose-response curves.

Tracheal segments were equilibrated to organ bath conditions for 1 h and regularly flushed with Krebs solution. After this period, viability was confirmed by contraction to 10^−4^ M acetylcholine; the bath was subsequently washed every 10 min with Krebs solution for a further 30 min. A diameter-force curve was then constructed to identify a reference diameter for contraction (to standardize mechanical history). Tracheal segments were contracted to KCl (80 mM) every 5 min at increasing lumen diameters. Passive and active force (total force − passive force) were determined at each diameter, until the contractile response reached a peak (Figure 1). Once the lumen diameter producing peak response to KCl was identified, the airway was returned to this diameter and adapted a further two times to KCl.

A cumulative dose-response curve for carbachol (CCh) was constructed (10^−9^ to 3 × 10^−5^ M). At the end of the dose-response curve, the organ bath was flushed with Krebs solution containing theophylline to completely remove muscle tone. A passive diameter-force curve was then constructed, from *D*_i_ to 1.2 *D*_i_, again in 5% increments. Force was recorded >30 s after each incremental diameter change, at which point stress-relaxation had subsided. At the end of the experiment, the length of the tracheal segment was measured under a dissecting microscope and airways were fixed in the organ bath (4% formaldehyde dissolved in Krebs solution).

### Airway Morphometry

Five-micrometer-thick transverse sections of the trachea were cut and stained with Masson’s Trichrome. The area and length of the ASM layer, total wall area and internal perimeter of the basement membrane (*P*_bm_) were measured using the newCast stereology software (Visiopharm, Hoersholm, Denmark).

### *In vivo* Assessment

In a separate group of mice (8-week-old offspring), the mechanical response of the airway wall to inflation/deflation was assessed *in vivo*. Information on the body weights of this group of mice including sex-specific changes in airway responsiveness have previously been described (Wang et al., 2018). Mice were anesthetized (i.p. 0.4 mg/g body weight of ketamine and 0.02 mg/g body weight of xylazine), tracheostomized, placed inside a whole-body plethysmograph, and mechanically ventilated at 400 breaths/min with a tidal volume of 10 ml/kg and 2 cmH_2_O positive end-expiratory pressure.

Respiratory system impedance (*Z*_rs_) was measured using a wave-tube system (Hantos et al., 1992) adapted for use in small animals (Peták et al., 1997; Sly et al., 2003) and a modification of the forced oscillation technique (Sly et al., 2003; Larcombe et al., 2013). During brief apneic periods, oscillatory signals between 4 and 38 Hz were delivered *via* a 1-m wave-tube to tracheostomized mice. Load impedance and lateral pressure at either end of the wave-tube were used to calculate *Z*_rs_ which was partitioned into the airway compartment (airway resistance, *R*_aw_ and inertance, *I*_aw_) and tissue compartment (tissue damping and tissue elastance) (Hantos et al., 1992).

Increases in transrespiratory pressure (*P*_rs_) were achieved by evacuating the air in the plethysmograph *via* a regulated vacuum source, while deflation occurred passively after removal of the pressure gradient. Mice were inflated and deflated between 0 and 20 cmH_2_O *P*_rs_ (~40 s) while the FOT signal was applied. The constant phase model was fit to *Z*_rs_ at 0.5-s intervals to calculate *R*_aw_ during the inflation-deflation maneuver (Larcombe et al., 2011). Three such maneuvers were performed, the first two to establish a volume history and the third for analysis.

### Analysis and Statistics

Active stress (g/mm^2^) was determined from force divided by ASM cross sectional area: thickness (ASM area/ASM length, mm) × length of tracheal segment (mm). Sigmoidal dose-response curves were fit to the data to estimate sensitivity to CCh, defined as the negative logarithm of the dose producing half maximal response (pD2). Passive stress (g/mm^2^) was determined from force divided by total wall cross sectional area: wall thickness (wall area/*P*_bm_, mm) × length of tracheal segment (mm). Forces used in the calculations were modified when calculating passive stress: since both sides of the tracheal segment act on the force transducer, the recording is likely double the physiological level and for this reason was halved. The analysis does however assume a flattened lumen which may not be completely true for a tracheal segment that contains stiff C-shaped cartilaginous rings at its anterior surface. Nonetheless, this correction does not affect comparisons between groups, only the absolute values reported. No correction was necessary for active stress since the ASM is only present on the posterior surface of the segment. Stiffness was calculated from the change in stress divided by the change in diameter strain (i.e., ∆stress/0.2).

For *in vivo* measurements, conductance was calculated from the inverse of *R*_aw_ (Wong et al., 2012), and subsequently converted into a global airway radius (radius = conductance^1/4^). Normalized radius-*P*_rs_ curves (radius/radius at 0 cmH_2_O *P*_rs_) were then constructed and used to calculate the storage modulus, the loss modulus, and the hysteresivity (loss/storage modulus) using well-established methods for assessment of cyclic forcing in nonlinear physiological systems (Oliver et al., 2010).

Graphical and statistical analyses were performed by SigmaPlot (13.0) and Prism version (7.02). Birth weights were compared by *t*-test. Two-way ANOVA was used to determine the effect of treatment and dose on active ASM stress, and the effect of treatment and sex on all other parameters. Data were transformed where necessary to ensure the assumptions of normality and homoscedasticity of variances for the parametric tests were satisfied. When data could not be normalized, equivalent non-parametric statistical analyses were used. All data are presented as mean ± SEM with ^*^p < 0.05 considered significant. *n* refers to number of offspring.

## Results

### Growth Outcomes

**Figure 2:**
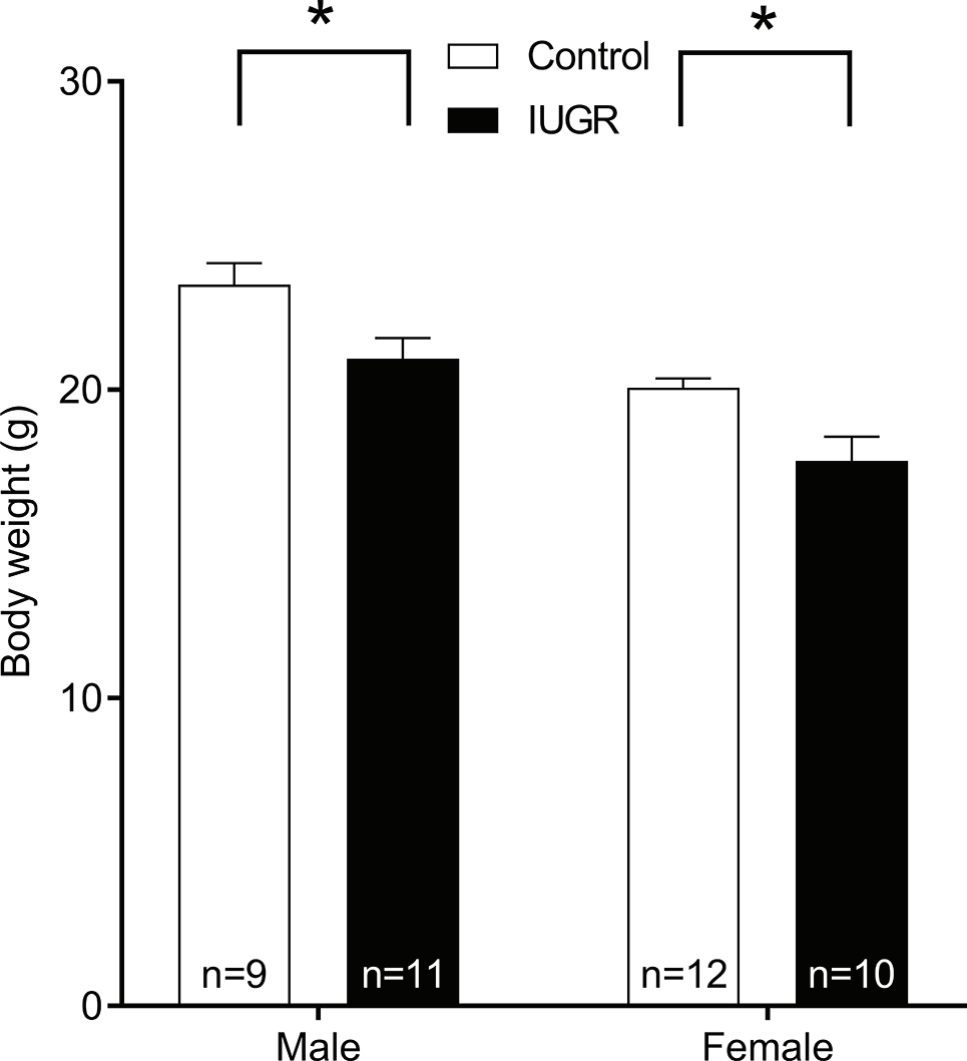
Body weight of Control and IUGR offspring at 8 weeks of age. Values are mean ± SEM. ^*^Significantly different from Control (*p* < 0.001). Control (white bar); IUGR, intrauterine growth restriction (black bar).

The IUGR offspring (*n* = 42) were lighter at birth compared with Control (*n* = 49) offspring (*p* = 0.03; unsexed) and remained lighter at 8 weeks of age (*p* < 0.001; Figure 2). Male offspring were also heavier compared with female offspring (*p* < 0.001). Maternal hypoxia had no effect on litter size (Control, 3.47 ± 0.34 pups; IUGR, 3.23 ± 0.28 pups; *p* = 0.599) or gestational period (Control, 19.88 ± 0.15 days; IUGR, 19.94 ± 0.006 days; *p* = 0.507). Tracheal *P*_bm_ (an index of airway size) was not affected by IUGR (*p* = 0.329; Table 1) or sex (*p* = 0.812).

**Table 1:** Airway size.

Control		IUGR	
Male (*n* = 9)	Female (*n* = 12)	Male (*n* = 11)	Female (*n* = 10)
2.649 ± 0.10 mm	2.859 ± 0.09 mm	2.749 ± 0.09 mm	2.716 ± 0.06 mm

*Values are mean ± SEM. IUGR, intrauterine growth restriction*.

### Active Airway Smooth Muscle Stress

**Figure 3:**
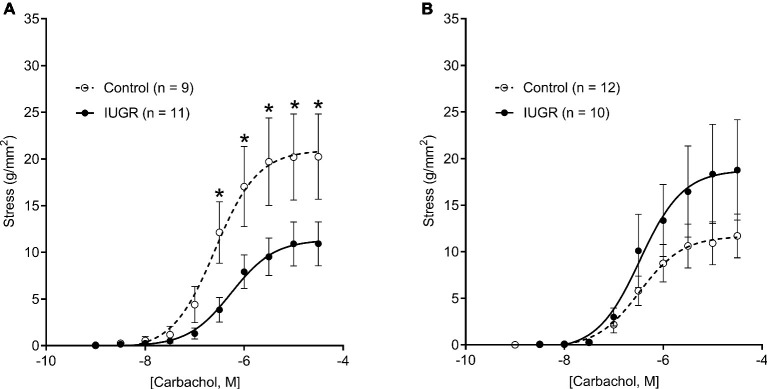
CCh-stress dose-response curves in males **(A)** and females **(B)** for Control and IUGR offspring. Values are mean ± SEM. ^*^Significantly different from Control (*p* < 0.05). CCh, carbachol; Control (white circles); IUGR, intrauterine growth restriction (black circles).

CCh-stress dose-response curves are shown in Figure 3. Compared with male Control offspring, active stress was reduced in male IUGR offspring at CCh doses ranging from 3 × 10^−7^ M to a maximal concentration of 3 × 10^−5^ M (*p* < 0.05; Figure 3A). There was no difference in active ASM stress between female groups (*p* = 0.738; Figure 3B). Sensitivity to CCh, as reflected by pD2, was not different between groups (Control male, 6.52 ± 0.07; Control female, 6.47 ± 0.07; IUGR male, 6.34 ± 0.07; IUGR female, 6.45 ± 0.11; *p* = 0.22) and was not affected by sex (*p* = 0.737).

### Passive Airway Stiffness *in vitro*

**Figure 4:**
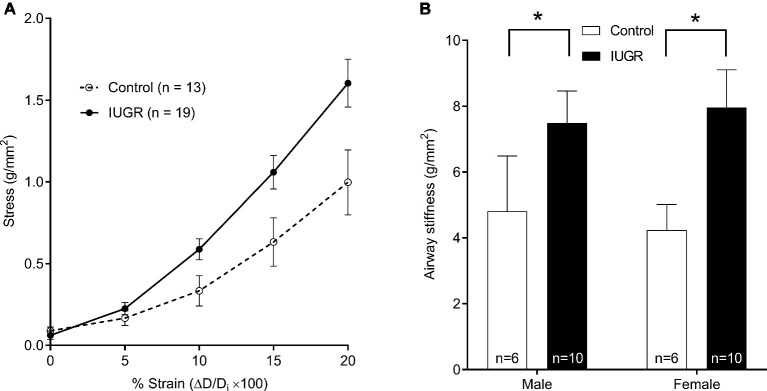
Relationship between wall stress and lumen diameter strain (sexes combined), where *D*_i_ was the initial diameter after mounting in the organ bath **(A)**. Passive airway stiffness in males and females for Control and IUGR offspring **(B)**. Values are mean ± SEM. ^*^Significantly different from Control (*p* < 0.05). Control (white circles/bars); IUGR, intrauterine growth restriction (black circles/bars).

Passive airway stiffness was calculated from changes in wall stress over the relative change in diameter strain (Figure 4A). Passive airway stiffness was greater in IUGR offspring compared with Control offspring (*p* = 0.003; Figure 4B). Passive airway stiffness was not affected by sex (*p* = 0.538).

### Airway Moduli and Hysteresivity *in vivo*

**Figure 5:**
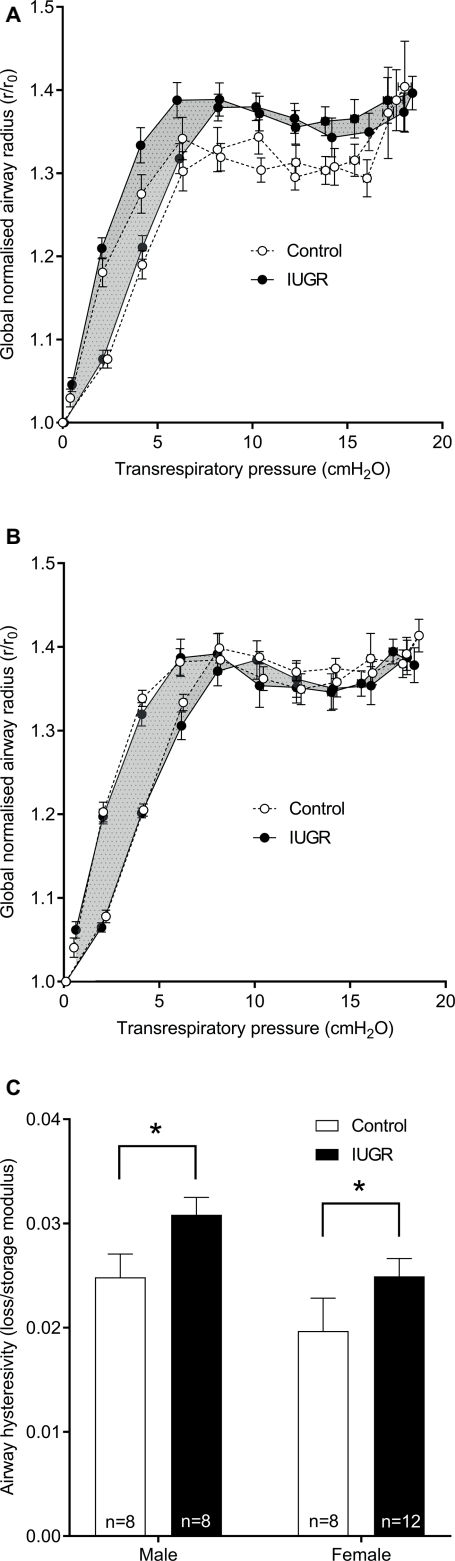
Global airway radius against transrespiratory pressure. Conductance was calculated from the inverse of resistance and subsequently converted into a global airway radius (radius = conductance^1/4^) normalized to initial radius at 0 cmH_2_O (*r*/*r*_0_). Inflationary and deflationary curves were plotted for males **(A)** and females **(B)**. In the IUGR group, the area between inflation and deflation curves is shaded for clarity. Airway hysteresivity for Control and IUGR offspring (**C**; loss/storage modulus). Values are mean ± SEM. ^*^Significantly different from Control (*p* < 0.05). Control (white circles/bars); IUGR, intrauterine growth restriction (black circles/bars).

From the global (normalized) airway radius-*P*_rs_ curves shown in Figures 5A,B, the compliant region of the curve is between 0 and 5 cmH_2_O, above which the airway is seen to stiffen. There was no difference in storage (*p* = 0.187) or loss moduli (*p* = 0.168) between groups. Hysteresivity was however increased in the IUGR group compared with Control (*p* = 0.016; Figure 5C) (note that findings were similar when analyzed as *R*_aw_ or conductance rather than global airway radius). Sex was also an independent predictor; loss modulus (*p* = 0.038) and hysteresivity (*p* = 0.018) were increased in males compared with females.

## Discussion

There is emerging data demonstrating that IUGR and low birth weight impact airway development and subsequent function, and in turn risk of developing obstructive airway disease in adulthood (Roseboom et al., 2003; Turner et al., 2010). Using our established mouse model of maternal hypoxia-induced IUGR (Wang et al., 2018), we generated offspring with low birth weight and demonstrated changes in the physiology of the airway that may account for changes in airway responsiveness observed in our previous investigation (Wang et al., 2018). Our results suggest that mechanical abnormalities of the airway wall develop after an *in utero* insult and could alter susceptibility for asthma development.

Our model of IUGR develops after a period of induced maternal hypoxia. Ultimately birth weight is a marker of adverse fetal development and many of the risk factors for IUGR (placental restriction and under nutrition) exert their effects through a hypoxic insult (Wickström, 2007; Belkacemi et al., 2010). Evidence from prenatal sheep studies indicate that partial pressure of oxygen in the fetal carotid artery is positively correlated with birth weight (Botting et al., 2012). The magnitude of birth weight restriction observed in the present study was small (~5%) and approximately half that of our earlier study (Wang et al., 2018). When comparing mean birth weight of IUGR animals to the Control group, growth-restricted animals are placed below the <32nd percentile group of the Control group, whereas the clinical definition of IUGR is birth weight <10th percentile (Peleg et al., 1998). The intervention was nonetheless sufficient to produce physiological abnormalities that persisted to the adult airway.

Other than ASM thickness, the force produced for a given cross sectional area of ASM, the definition of stress or generically “contractility,” is expected to impact airway narrowing capacity to an applied agonist. Force or stress is only feasibly assessed *in vitro*, specifically in excised segments of airway or strips of muscle preserved in organ bath chambers. One such *in vitro* study examined ASM responses from guinea pigs exhibiting hyperresponsiveness *in vivo,* and demonstrated an increase in pressure generation to contractile agonist, which was not accounted for by changes in ASM thickness, consistent with increased ASM contractility (Ishida et al., 1990). Hyperresponsiveness *in vivo* is however not always accompanied by increased ASM contraction *in vitro* (Ishida et al., 1990), with some studies even reporting a reduced contractility of ASM from hyperresponsive animals (Turner et al., 2002). Inconsistencies between *in vivo* and *in vitro* findings on airway reactivity reflect complexity in the physiology of airway narrowing, which is determined by a balance of forces produced by the ASM layer, mural loads, and airway-lung interdependence in the dynamic breathing environment (Harvey and Lutchen, 2013). Sexual dimorphism in airway responsiveness is also a consideration, as we demonstrated in IUGR offspring where males were hyporesponsive and females hyperresponsive compared with Control offspring (Wang et al., 2018).

The present data on ASM contraction *in vitro* provides an explanation for hyporresponsiveness in male IUGR offspring *in vivo*. Maximal contractility of the ASM was reduced relative to male Control offspring and should intuitively decrease ASM shortening and luminal narrowing to bronchial challenge *in vivo*. In comparison, there were no clear changes in ASM contractility in female IUGR offspring, indicating that active ASM properties are not affected by IUGR. The effect of IUGR on smooth muscle contraction has been previously assessed in the vascular system. Coronary arterial segments isolated from sheep subject to IUGR late in gestation were more responsive to vasoconstrictors than Control group (Bubb et al., 2007). In contrast, carotid arteries of neonatal rats showed blunted contractions following maternal hypoxia, which the authors proposed was not due to changes in muscle thickness (Williams et al., 2005). None of these studies identified the sex of the offspring, and as such we were unable to conclude if sex impacted arteries similar to our findings on the murine airway.

Beyond establishing a mechanism for hyporesponsiveness in male IUGR offspring *in vivo*, the data generated from the present study do not provide information on why the contractility of the ASM layer was herein modified. Given that the experimental intervention was applied during the fetal period, known maturational changes in ASM contraction are relevant. Sparrow and Mitchell compared contractility of tracheal ASM from fetal, young and mature pigs (Sparrow and Mitchell, 1990). While contractility varied between time points, differences were minimized after accounting for myosin content that increased with maturity. Ontogenetic changes in myosin light chain kinase content are observed in guinea-pig airways (Chitano et al., 2004), which offers an explanation for increased airway reactivity in early life (Montgomery and Tepper, 1990). An effect of IUGR on contractile filament expression, myosin light chain kinase, or other key components of the contractile cycle would impact force production. Changes extrinsic to the ASM cell are also possible; we report increased proportion of extracellular matrix within the ASM layer of subjects with fixed airflow limitation (Jones et al., 2016). If the proportion of matrix to muscle cells within the ASM layer were altered by IUGR, the amount of contractile tissue contained within a given cross sectional area of ASM layer will change and be reflected in our measures of contractility. Matrix changes further impact the micro-mechanical environment of ASM cells which through mechanosensation pathways determine contractility at a cellular level (An et al., 2009). Future investigations should focus on phenotyping the composition of the ASM layer and contractility at a tissue and cellular level.

Due to advancements in our understanding on the physiological behavior of ASM, particularly contraction under conditions of dynamic forces accompanying breathing, it is now prudent to examine properties beyond simple changes in ASM thickness and contractility when attempting to reveal mechanisms for abnormalities in airway responsiveness. Increased airway stiffness has been documented in subjects with asthma (Brackel et al., 2000), possibly due to gross changes in wall structure (Ward et al., 2001) and/or increased passive tension of the ASM layer (Chin et al., 2012). The implications of a stiffer airway wall in asthma is that protective effects of breathing maneuvers including deep inspiration may be blunted, which through distension of the airway wall and ASM layer, normally, reduces ASM force production (Noble et al., 2007) and in turn expands lumen caliber (West et al., 2012). Similar to what is proposed in asthmatic subjects, the IUGR group exhibited increased airway stiffness *in vitro*. Findings are unlikely due to changes in tissue bulk, since we previously observed no changes in airway wall structure when sampling systematically through the IUGR-affected murine lung (Wang et al., 2018). Intrinsic changes to the ASM layer are possible, which is modifiable without apparent changes in structure (Seow, 2013). Changes in cartilage stiffness are also worth considering, particularly in our system where cartilage and ASM layers are arranged in parallel, such that cartilage may contribute in no small way to the reported stiffness values.

At an airway level, the net effect of changes in ASM contractility and airway stiffness will determine airway responsiveness in IUGR mice *in vivo*. In male IUGR offspring exhibiting hyporesponsiveness *in vivo* (Wang et al., 2018), reduced contractility may outweigh the detrimental effects of increased stiffness and/or there may be protective effects in preventing collapse (Noble et al., 2002). In comparison, female IUGR offspring do not show the same attenuation in ASM contractility and, in this scenario, increased stiffness is accompanied by hyperresponsiveness *in vivo* (Wang et al., 2018). Our observations are consistent with human population studies that report a greater susceptibility of adult females to asthma development (Schatz and Camargo, 2003), an association that may begin *in utero*.

A limitation of the present study is the use of the trachea to model *in vivo* lung function which is governed by the behavior of intra-parenchymal airways. Tracheal ASM is a commonly studied tissue due to its relative thickness and ease of dissection. Contractile properties of the tracheal ASM in asthma are consistent with studies performed on large bronchi (Ijpma et al., 2015). There is however some evidence from horses with heaves (an innate model of asthma) to suggest that in the context of disease, peripheral airways behave differently to more proximally located airways (Matusovsky et al., 2016). Moreover, the conclusions drawn from our earlier biological and mathematical modeling study attributed increased heterogeneity of lumen caliber to an anatomical variation in airway compliance (Wang et al., 2017). Assessment at a single location may therefore not be representative of integrated function *in vivo*.

The above reductionist *in vitro* approach provides information at an airway level on how tissue changes contribute to previously observed changes in respiratory function after IUGR. In separate experiments, we examined how apparent increases in airway stiffness within the IUGR group *in vitro* would manifest more globally *in vivo*. Mechanical properties of the bronchial tree were assessed from the inverse of airway resistance (conductance) to the one-quarter power, approximating changes in global lumen radius. That is, the bronchial tree was represented as a single lumen radius, an approach which itself carries numerous assumptions, albeit different assumptions to studies conducted *in vitro*. After normalization to baseline caliber, IUGR animals exhibited an increase in hysteresivity, but no change in storage or loss modulus. In other murine models, changes in tissue (lung) hysteresivity have been observed following fibrotic disease (Pillow et al., 2001). The lack of an effect of IUGR on airway storage modulus (equivalent to *in vitro* measurements of stiffness) may be due the aforementioned heterogeneity in compliance predicted in our earlier study (Wang et al., 2017). *In vivo*, these data suggest that the dominant effect is an increase in airway viscosity after IUGR, which may also contribute to impaired distension of the airway wall to dynamically applied loads. There is of course the possibility that changes in airway stiffness identified *in vitro* included some component of viscous load, although this is unlikely as measurements were performed under quasistatic conditions after stress-relaxation forces had subsided. Finally, an interesting observation was the clear sex differences in airway loss modulus which was increased in males compared with females. Such differences may further contribute to sex differences in respiratory pathophysiology.

In conclusion, the present study demonstrates mechanical abnormalities of the ASM layer and airway wall in adult mice after IUGR. Such abnormalities are expected to alter susceptibility for asthma development, in a sex-dependent manner, which has implications for our understanding of the pathogenesis of asthma and early life preventative measures.

## Data Availability

All datasets generated for this study are included in the manuscript and/or supplementary files.

## Ethics Statement

The animal study was reviewed and approved by the Telethon Kids Institute Animal Ethics Committee.

## Author Contributions

PN, AL, and KW designed the study. DK and KW were involved in data collection. PN and KW drafted the manuscript. All authors were involved in data analysis and interpretation and revised the manuscript critically for important intellectual content and approved the final version of the manuscript.

### Conflict of Interest Statement

The authors declare that the research was conducted in the absence of any commercial or financial relationships that could be construed as a potential conflict of interest.
